# Skin Complications of Orthopedic Procedures and Devices

**Published:** 2018-12

**Authors:** Zahra AZIZIAN, Zeinab HESAMI, Parvin MANSOURI, Adel EBRAHIMPOUR, Bahamin ATTAR, Reza CHALANGARI

**Affiliations:** 1. Skin and Stem Cell Research Center, Tehran University of Medical Sciences, Tehran, Iran; 2. Dept. of Orthopedic, Shahid Beheshti University of Medical Sciences, Tehran, Iran; 3. Dept. of Dermatology, Iran University of Medical Sciences, Tehran, Iran; 4. Kassir Dermatology, Dallas, Texas, USA

**Keywords:** Skin complication, Orthopedic procedures, Orthopedic devices

## Abstract

**Background:**

Knowledge of skin complications and contributing factors in orthopedic patients is important for design and development of preventive approaches. Therefore, this study was designed to assess skin complications in orthopedic patients.

**Methods:**

In this case-series study, 126 orthopedic patients referred to Rasoul-e-Akram and Bahman hospitals from 2012 to 2016 with skin complications were analyzed. The adverse effects were assessed with respect to type and contributing factors. Fisher’s exact test, Chi-square, and independent sample t-test were performed to assess the associations between skin complications and other variables.

**Results:**

Skin complications in orthopedic patients included infections in 33 (26.1%) cases and hypersensitivity reactions in 88 (40%) cases. In total, 66 (55%) cases of fracture and 35 (29.2%) cases of cellulitis were detected, while the remaining cases involved complications such as disc herniation, nerve involvement, and osteoarthritis-related arthroplasty. Severe reactions presenting as toxic epidermal necrolysis were observed in 3 patients, 2 of whom died eventually. Age and gender were not related to the type of skin complications (*P*>0.05).

**Conclusion:**

Complications due to orthopedic treatments were not common. However, since the disease may become fatal on certain occasions, patients should receive more attention from physicians and nurses.

## Introduction

Surgical procedures are associated with different complications. Some of these complications may occur in patients, despite their rare occurrence and major preventive measures applied by physicians. Similarly, complications associated with orthopedic surgeries are still reported, despite major improvements in this area. Overall, all surgical procedures, regardless of their scope, may cause insignificant to serious (e.g., fatal complications) complications in patients (1, 2).

Postoperative complications can be general or specific to the type of surgical procedure; management of these complications is dependent on the patient’s medical history. Postoperative fever, atelectasis, embolism, wound infection, and deep vein thrombosis is the most common general complications in patients. The highest incidence of these complications is generally reported within 1 to 3 postoperative days. Nevertheless, specific complications may occur in the early postoperative period, a few days after surgery, during the postoperative period, and in the late postoperative period, respectively (3, 4).

Considering the associated morbidities and pain among patients, dedicated surgeons are usually troubled by the development of postoperative complications. In fact, all surgical procedures, even the simplest ones, are associated with an unending list of complications. These complications can be due to the imprecise evaluation of patients, type of implant, or the surgeon’s decisions and practice.

On the other hand, the causes of complications may be multifactorial or even unknown in some cases. Overall, infections are recognized as possible postoperative complications and are among the most serious complications associated with orthopedic surgeries. Moreover, tissue infections may occur at different ages among male and female patients (5). In addition, skin infections are prevalent occurrences, accounting for 5%–10% of complications in surgical settings (6).

Orthopedic surgery devices and materials are generally well tolerated by patients. Infections, mechanical problems, and allergic reactions (or hypersensitivity) are common patient complaints after orthopedic surgeries. The allergic reactions include cutaneous changes (such as eczema), pain, recurrent effusion, delay in wound healing, and implant loosening.

Unlike cutaneous metal contact allergies, which have high incidence rates, implant-associated allergies are quite rare. Nevertheless, there is little epidemiological information regarding the occurrence of these reactions (7). Such skin problems may not only result in the deterioration of patient’s condition but also lead to reoperation and other side effects (8–10).

Post operation complication of orthopedic surgery (e.g. fever and surgical site infection) could occur in approximately 2%–5% of the patients especially in the young adult (11). Although there are not any reports regarding the prevalence of these complications among the Iranian patinas and its cost on the health system, in the USA a financial cost of treatment is up to $10 billion annually (12). In addition, surgical site infection after orthopedic surgeries leads to increased morbidity, mortality, extended hospital in-patient stays, and economic burden to the hospital resources (13). Accordingly, it is better to prevent rather than treat infections and hypersensitivity reactions, resulting from orthopedic procedures and devices (14).

For this matter, knowledge of skin complications and contributing factors in orthopedic patients is important for the design and development of preventive approaches. Accordingly, the present study designed to assess these complications.

## Methods

This retrospective descriptive study was conducted during 2013–2016. Among 1820 cases (including outpatient and hospitalized patients), referred to the orthopedic trauma centers of Rasoul Akram and Bahman Hospitals, Tehran, Iran; we selected 126 patients, subsequently referred to a dermatologist by an orthopedic surgeon due to skin complications.

The type of skin complications and contributing factors were assessed among patients. Skin complications were mostly diagnosed based on the clinical symptoms detected by a dermatologist; in few cases, definitive diagnosis was made by skin sampling. The exclusion criteria were incomplete data and known skin diseases. Well-trained and experienced specialists diagnosed the patients.

Written consents were obtained from the participants, and the local Ethics Committee approved the study.

Data collection was carried out, using the available medical records and data in the checklists. Fisher’s exact test, independent sample t-test, and Chi-square were performed to assess the associations between skin complications and other variables. For statistical analysis, SPSS ver. 13.0 (Chicago, Illinois, USA) was used (significance level, *P*<0.05).

## Results

The mean age of the participants was 57.1±12.6 yr. Moreover, 52 (41.2%) and 74 (58.7%) patients were female and male, respectively. Overall, 66 cases of fracture and 35 cases of cellulitis were detected. The remaining cases included complications, such as disc herniation, nerve involvement, and osteoarthritis-related arthroplasty. The patients’ conditions are shown in Table 1.

**Table 1:** Demographic Data of patients

***Variable***	***N(%)***
Gender	
Male	74 (58.7)
Female	52(41.2)
Site of hospital reference	
Orthopedic emergency room	10 (8.3)
Orthopedic ward	67 (55.8)
Intensive care unit	5 (4.2)
Orthopedic clinic	38 (31.7)
Underlining disease	
Fracture cases	66 (52.3)
Arthroplasty	28 (43)
Upper extremities	16 (24)
Lower extremities	14 (21)
Open fracture	8 (12)
Cellulitis	35 (27.7)
Disc herniation	4 (3.1)
Tendonitis	11 (8.7)
Osteoarthritis-related arthroplasty	10 (7.9)

Skin complications in orthopedic patients included infections in 33 (26.1%) cases, hypersensitivity reactions in 88 (73.3%) cases, and 5 (4.1%) cases with other complications. Local skin reactions were observed as irritant and allergic contact dermatitis with erythema, pruritus, and less frequently, bullous lesions (Table 2). Orthopedic implants and bandages were responsible for most cases of local contact dermatitis. Systemic skin reactions due to drugs are presented in Table 3.

**Table 2:** Local skin reaction to most common orthopaedic devices

***Reaction***	***Percent***	***Number***
Surgical Tapes	12.9	8
Corn and callus removal tapes	8	5
Orthopedic Casts	20.9	13
Orthopedic Implants (plates and screws)	22.5	14
Topical Herbal Medicines	8	5
Wrist, knee bandages	27.4	17

**Table 3:** Generalized Drug Reactions to most common orthopaedic drug prescription

***Drug***	***Eruption***				
	***Urticaria (%)***	***Stevens /TEN (%)***	***Maculopapular Eruption (%)***	***Fixed Drug Eruption (FDE) (%)***	***Vasculitis (%)***
NSAID	4(15.3)	2(7.6)	2(7.6)	1(7.6)	1(7.6)
Antibiotics					
Cephazolin		1(3.8)	2(7.6)		
Cephalexin		1(3.8%)	3(11.5)		
Ceftriaxone			2(7.6)		
Ciprofloxacin					2(7.6)
Alendronate	1(3.8)		1(3.8)		
Cartilage repair supplement	1(3.8)		1(3.8)		1(3.8)

Severe skin reactions, presenting as toxic epidermal necrolysis, were observed in 3 cases, 2 of whom died eventually. One patient was an 18-yr-old girl (with a prophylactic injection of cefazolin before ulnar fracture surgery), who died due to sepsis. The second patient was an 84-yr-old man (with diclofenac injection for discopathy pain relief), who died due to massive gastrointestinal bleeding, resulting from severe mucosal erosion. The third patient died due to the use of piroxicam for back pain relief. In addition, cephalexin for foot cellulitis led to Stevens-Johnson syndrome in 1 case. No significant correlation was observed between the underlying cause of admission and skin reactions (*P*=0.6).

Other complications included skin lacerations after implanting plates in 2 patients and depigmentation after intralesional corticosteroid injection for tendonitis in 3 patients. Cellulitis infection was observed in 33 (26.1%) patients. All these cases occurred in hospital-admitted patients. The most common site of laceration was the surgical site in 19 patients, with 8 patients experiencing intravenous catheter infections.

The most common causes of bacterial infection were *Staphylococcus* species (86%) and *Pseudomonas aeruginosa* (14%). Six patients had intertrigo with Candida and tinea infections in the inguinal region. All intertrigo patients underwent hip arthroplasty surgery. However, there was no significant correlation between the underlying cause of admission and infection rate (*P*=0.7). In addition, age and gender were not related to the type of skin complication in patients (*P*=0.6).

## Discussion

In this study, skin complications were assessed in orthopedic patients. The skin complications of orthopedic procedures and devices are presented in 3 main categories, including infections, hypersensitivity reactions, and less common adverse effects. When the implants are located on the target site, successful biointegration requires colonization of a highly reactive implant surface by host cells (15).

Bacteria such as *Staphylococcus* species may attach to metallic or polymeric devices and colonize the implant surface instead of the host cells. Once attached, these bacteria can develop a biofilm and undergo phenotypic changes, which make them somewhat resistant to the host’s immune responses and even antibiotics (15–17). Infections at or near surgical incisions within 30 d of an operative procedure are known as surgical site infections, which may present as skin infections (0.5%–2%) (18). The most common contributing bacteria are skin flora, such as coagulase-negative *Staphylococci* (19, 20).

Occasionally, other less common bacteria may be seen in some patients, especially those with immunodeficiency or underlying diseases (21–23). These infections are usually treatment-resistant, and responses to common antibiotics are less expected (24, 25). In more procedures complex, half of infected patients may require reoperation for wound debridement or sometimes skin flap closure (26).

Use of broad-spectrum antibiotics may be optionally indicated to reduce the resistance and recurrence of skin infections after orthopedic procedures (27, 28). In severe cases, removal of external devices may be necessary (29). In this study, the infections were the most common adverse effects and were more common in diabetic patients. Allergic reactions to implants, fixators, other orthopedic devices, or bone cement components are among other skin complications (30, 31). Nickel, chromium, and benzoyl peroxide are the major metallic etiologies for these allergic reactions (32, 33). Allergic reactions were the second most prevalent complication after infections in our study.

The common symptoms of skin complications include swelling, pain, inflammatory skin reactions, implant loosening, and occasionally fistula formation (33–35). These hypersensitivity reactions may cause orthopedic implant failure, leading to reoperation (29, 30). Hypersensitivity reactions may be seen in up to one-fourth of patients undergoing orthopedic surgeries (36, 37).

The need for reoperation and removal of orthopedic devices (or replacement with less allergenic devices) may be indicated in severe cases (38, 39). The effectiveness of local antiallergic components has not been evaluated yet. Trauma injuries, especially in pediatric patients and those requiring long-term casting, are other less common skin complications (40, 41, 42). However, skin can tolerate cast immobilization even for a prolonged duration (41). These types of skin complications are more preventable than the previously mentioned skin complications in orthopedic patients (43,45, 46). In addition, spontaneous improvement is an expected consequence in the majority of cases.

Systemic drug reactions in orthopedic surgeries are similar to other surgeries. The most routinely prescribed drugs in orthopedic clinics are nonsteroidal anti-inflammatory drugs (NSAIDs) (50). Although skin eruption due to NSAIDs is commonly mild, severe conditions, such as toxic epidermal necrolysis and Stevens-Johnson syndrome, may occur. In this study, the majority of negative reactions to NSAIDs were urticarial eruptions, although vasculitis (a rare skin reaction to NSAIDs) has been reported in the literature (51). Various bacteria have been observed in many studies to cause infection surgical site (44). Systemic antibiotic prophylaxis in orthopedic implant surgeries is the standard practice, used in the past 3 decades. Cephazolin is a commonly prescribed drug, and urticaria and maculopapular eruptions are the most common reactions; fatal reactions have been also reported in other studies (52). In this study, four patients had TEN; using this kind of drugs in any surgery can cause such side effects. Three per 10000 patients in orthopedic wards can experience such drug reactions, and careful attention must be paid to the high risk of TEN with NSAIDs and Antibiotics.

Generally, scattered and limited studies have been done in this area, whose results are mostly consistent with our study, though with certain differences due to the type of study, type of treatment, etc. The prophylactic use of antibiotics was evaluated, (47) or examined the difference between the upper and lower extremity infections in Germany (48) and a study (49) investigated the difference between the position of the fracture and postoperative infection.

There are some other skin complications associated with orthopedic procedures, such as hypopigmentation after local corticosteroid injection to control the joint and tendon inflammation. Overall, this complication is quite rare (estimated risk, <1%), and steroid injections may result in skin atrophy or hypopigmentation (50). On the other hand, we can reduce the risk of subcutaneous fat atrophy and hypopigmentation by using soluble and potent steroids. Overall, low-solubility steroids (e.g., triamcinolone acetonide) are suggested for deep structures (e.g., knee), while high-solubility steroids (e.g., betamethasone and dexamethasone) are administered preferably in soft tissues (e.g., carpal tunnel and tendon sheath) (53).

## Conclusion

However, there are skin complications that are not so common in orthopedic patients but remain important and life-threatening, and thus should be considered by physicians. Skin complications of orthopedic procedures and devices are common causes of operation failure. These may be more serious, such as skin infections, or less important, such as traumatic injuries. Knowledge of these adverse effects is essential to prevent them and improve the postoperative outcomes in patients.

## Ethical considerations

Ethical issues (Including plagiarism, informed consent, misconduct, data fabrication and/or falsification, double publication and/or submission, redundancy, etc.) have been completely observed by the authors.
